# High CO_2_ Triggers Preferential Root Growth of *Arabidopsis thaliana* Via Two Distinct Systems Under Low pH and Low N Stresses

**DOI:** 10.1093/pcp/pcu001

**Published:** 2014-01-30

**Authors:** Takushi Hachiya, Daisuke Sugiura, Mikiko Kojima, Shigeru Sato, Shuichi Yanagisawa, Hitoshi Sakakibara, Ichiro Terashima, Ko Noguchi

**Affiliations:** ^1^Department of Biological Sciences, Graduate School of Science, The University of Tokyo, 7-3-1 Hongo, Bunkyo-ku, Tokyo, 113-0033 Japan; ^2^RIKEN Center for Sustainable Resource Science, 1-7-22 Suehiro-cho, Tsurumi-ku, Yokohama, Kanagawa, 230-0045 Japan; ^3^Biotechnology Research Center, The University of Tokyo, 1-1-1 Yayoi, Bunkyo-ku, Tokyo, 113-8657 Japan

**Keywords:** Auxin, Cytokinin, High CO_2_, Low nitrogen, Low pH, Root to shoot ratio

## Abstract

Biomass allocation between shoots and roots is an important strategy used by plants to optimize growth in various environments. Root to shoot mass ratios typically increase in response to high CO_2_, a trend particularly evident under abiotic stress. We investigated this preferential root growth (PRG) in *Arabidopsis thaliana* plants cultivated under low pH/high CO_2_ or low nitrogen (N)/high CO_2_ conditions. Previous studies have suggested that changes in plant hormone, carbon (C) and N status may be related to PRG. We therefore examined the mechanisms underlying PRG by genetically modifying cytokinin (CK) levels, C and N status, and sugar signaling, performing sugar application experiments and determining primary metabolites, plant hormones and expression of related genes. Both low pH/high CO_2_ and low N/high CO_2_ stresses induced increases in lateral root (LR) number and led to high C/N ratios; however, under low pH/high CO_2_ conditions, large quantities of C were accumulated, whereas under low N/high CO_2_ conditions, N was severely depleted. Analyses of a CK-deficient mutant and a starchless mutant, in conjunction with sugar application experiments, revealed that these stresses induce PRG via different mechanisms. Metabolite and hormone profile analysis indicated that under low pH/high CO_2_ conditions, excess C accumulation may enhance LR number through the dual actions of increased auxin and decreased CKs.

## Introduction

Atmospheric CO_2_ concentrations have been rapidly rising since the Industrial Revolution. Current evidence suggests that increased CO_2_ levels at the end of this century will have major impacts on plant growth and development (Taub 2010). In general, high CO_2_ concentrations increase photosynthetic carbon (C) fixation rates, which in turn furnish additional C substrates and stimulate plant growth (Ainsworth and Rogers 2007, Leakey et al. 2009). This enhanced growth often occurs unequally in shoots and roots (Rogers et al. 1992, Stulen and den Hertog 1993, Rogers et al. 1996). Root to shoot mass (R/S) ratios typically increase in response to high CO_2_, although ratios are variable depending on species and environmental conditions (Rogers et al. 1996, Sigurdsson et al. 2001, Ainsworth and Long 2005, de Graaff et al. 2006). Several meta-analyses have suggested that such preferential root growth (PRG) is more pronounced under abiotic stresses such as high light and nutrient or water deficiencies (Bazzaz 1990, Stulen and den Hertog 1993, Rogers et al. 1996). Plants exposed to abiotic stresses often accumulate sugars, possibly because of growth inhibition (i.e. decline in conversion of C substrates to structures) (Couée et al. 2006, Wingler and Roitsch 2008). Under high CO_2_ and abiotic stress conditions, excess C accumulation may favor root growth over that of photosynthetic organs. This PRG contributes to maintenance of the balance between C and other nutrients at the whole-plant level (Van Noordwijk and de Willigen 1987, Rogers et al. 1996).

R/S ratios vary depending on inorganic nitrogen (N) availability in soils and N status in plants (Scheible et al. 1997, Zhang et al. 1999). Scheible et al. (1997) have demonstrated that nitrate accumulation in shoots suppresses root growth. In addition, increases in amino acid and soluble protein concentrations in plant tissues are related to decreases in R/S ratios (Andrews et al. 2006, Walch-Liu et al. 2006, Pellny et al. 2008). Consequently, R/S ratios are systemically regulated by plant N status. Recent evidence indicates that nitrate assimilation in leaves is significantly suppressed under high CO_2_ conditions in C_3_ plants (Bloom et al. 2010). High CO_2_ stimulates plant growth, leading in turn to rapid consumption of soil N (Lambers et al. 1995), and organic N concentrations often decrease in plants under prolonged CO_2_ enrichment (Taub and Wang 2008, Bloom 2009). PRG at high CO_2_ levels may thus be related to decreases in N availability and changes in plant N status.

Using *Arabidopsis thaliana* and tobacco, reverse genetics approaches have provided evidence that plant hormones, particularly auxin and cytokinin (CK), are associated with regulation of R/S ratios. For example, mutants or transformants deficient in CK signaling or biosynthesis show repressed shoot growth and enhanced root growth (e.g. Werner et al. 2001, Miyawaki et al. 2006), and root-specific reduction of CK by increased expression of cytokinin-degrading cytokinin oxidase (*CKX*) in roots enhances root growth without affecting shoot growth (Werner et al. 2010). With respect to auxin, it is widely accepted that this hormone is a positive regulator of lateral root (LR) formation. Genetic defects in auxin signaling, transport or biosynthesis significantly impair LR formation (Péret et al. 2009). Recent studies have revealed that excess sugar application induces auxin biosynthesis via the phytochrome-interacting factor (PIF)-related pathway (Lilley et al. 2012, Sairanen et al. 2012). Lilley et al. (2012) found that sucrose application promotes auxin transport from shoots to roots. Because shoot-derived auxin can enhance LR growth (Reed et al. 1998, Bhalerao et al. 2002), auxin may act as a long-distance signal triggering PRG at high CO_2_ concentrations. Auxin is suggested to reduce biosynthesis of *trans*-zeatin (*t*Z)-type CKs by down-regulating *N*^6^-(Δ^2^-isopentenyl) adenine (iP) nucleotide hydroxylases in roots (Nordström et al. 2004, Takei et al. 2004). Consequently, auxin transported into roots may cause PRG partly by decreasing CK action. In contrast, CK negatively regulates polar auxin transport via PIN-formed (PIN) proteins and auxin-dependent enhancement of LR growth (Laplaze et al. 2007, Fukaki and Tasaka 2009, Růžička et al. 2009, Marhavý et al. 2011). Thus, CK, auxin and their interaction may play a crucial role in regulation of R/S ratios under high CO_2_ conditions.

Biomass allocation between shoots and roots is a fundamental strategy used by plants to optimize growth in fluctuating environments. To predict R/S ratio regulation in the upcoming ‘high CO_2_ world’, elucidation of the regulatory mechanisms operating under such conditions is therefore valuable. The aim of this study was to clarify factors responsible for PRG in response to high CO_2_. We used *A. thaliana* accession Col-0 as a study material because it is easy to analyze biomass allocation with rosette plants, and numerous Col-0 mutants are available. Plants were grown under ambient CO_2_ [390 parts per million by volume (ppmv)] or high CO_2_ (780 ppmv) with or without abiotic stresses. We subjected plants to low pH or low N stress because both stresses are important in agricultural and ecological fields (Sawaki et al. 2009, Krapp et al. 2011). Under each stress condition, high CO_2_ led to PRG in *A. thaliana*. We expected that these two stresses induced PRG differently, because low pH, unlike low N stress, does not cause N deficiency (Lang and Kaiser 1994, Ruan et al. 2007, Watanabe et al. 2010, Krapp et al. 2011). The mechanisms underlying PRG were thus examined by genetically modifying the CK level, C and N status and sugar signaling, by performing sugar application experiments and by determining primary metabolites, plant hormones andexpression of related genes. Our results demonstrate that two distinct systems trigger PRG under low pH and low N stress conditions at high CO_2_.

## Results

### Effects of high CO_2_ and abiotic stresses on preferential root growth

**Fig. 1:**
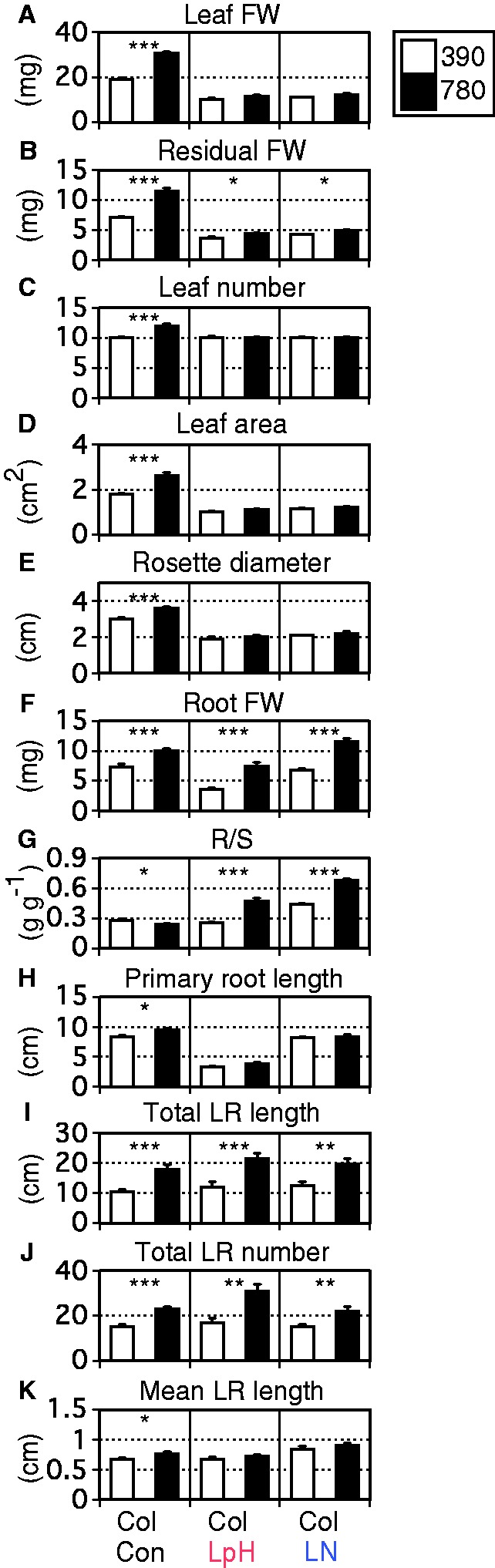
Effects of abiotic stresses and high CO_2_ on (A) leaf FW, (B) residual above-ground FW, (C) leaf number, (D) leaf area, (E) rosette diameter, (F) root FW and (G) R/S ratio of 14 d *Arabidopsis thaliana* Col-0 (Col) plants (i.e. 19-day-old plants) (*n* = 6–8) and (H) primary root length, (I) total LR length, (J) total LR number and (K) mean LR length of 10 d Col-0 plants (i.e. 15-day-old plants) (*n* = 10–25). White and black bars denote ambient (390 ppmv) and high (780 ppmv) CO_2_ conditions, respectively. Con, LpH and LN correspond to control, low-pH and low-N media, respectively. Student’s *t*-test was conducted (*P < 0.05; **P < 0.01; ***P < 0.001). Vertical bars represent the SEM.

We first examined growth of Col-0 plants cultivated for 14 d after transfer to control and stress conditions (14 d plants, i.e. 19-day-old plants). At this age, growth differences were obvious between ambient and high CO_2_ treatments. High CO_2_ stimulated growth even under abiotic stress conditions (Fig. 1), but manifestation of this growth varied depending on conditions. Leaf FW, residual above-ground FW, leaf number, leaf area and rosette diameter were greatly increased by high CO_2_ under control conditions, but only slightly enhanced under high CO_2_ and abiotic stress conditions (Fig. 1A–E). On the other hand, root FW was consistently increased by high CO_2_ under all conditions (Fig. 1F). High CO_2_ consequently resulted in PRG under abiotic stress conditions (Fig. 1G). In 21 d plants (i.e. 26-day-old plants), similar responses of R/S ratios to CO_2_ were observed under control and low pH conditions (Supplementary Fig. S1A–C).

To gain detailed information on PRG, root morphology was then quantitatively analyzed using vertically grown 10 d Col-0 plants (i.e. 15-day-old plants) (Fig. 1H–K). The 10 d treatment was sufficient to generate significant morphological changes in roots in response to increased CO_2_ levels. Under all conditions, primary root length was marginally increased by high CO_2_ (Fig. 1H), while total LR length was greaty stimulated (Fig. 1I). Total LR length was calculated by multiplying the total LR number by the mean LR length. Increases in total LR length were mainly due to increases in total LR number (Fig. 1J, K). PRG is thus closely associated with enhanced LR number.

### Effects of CK deficiency and excess soluble C on PRG

**Fig. 2:**
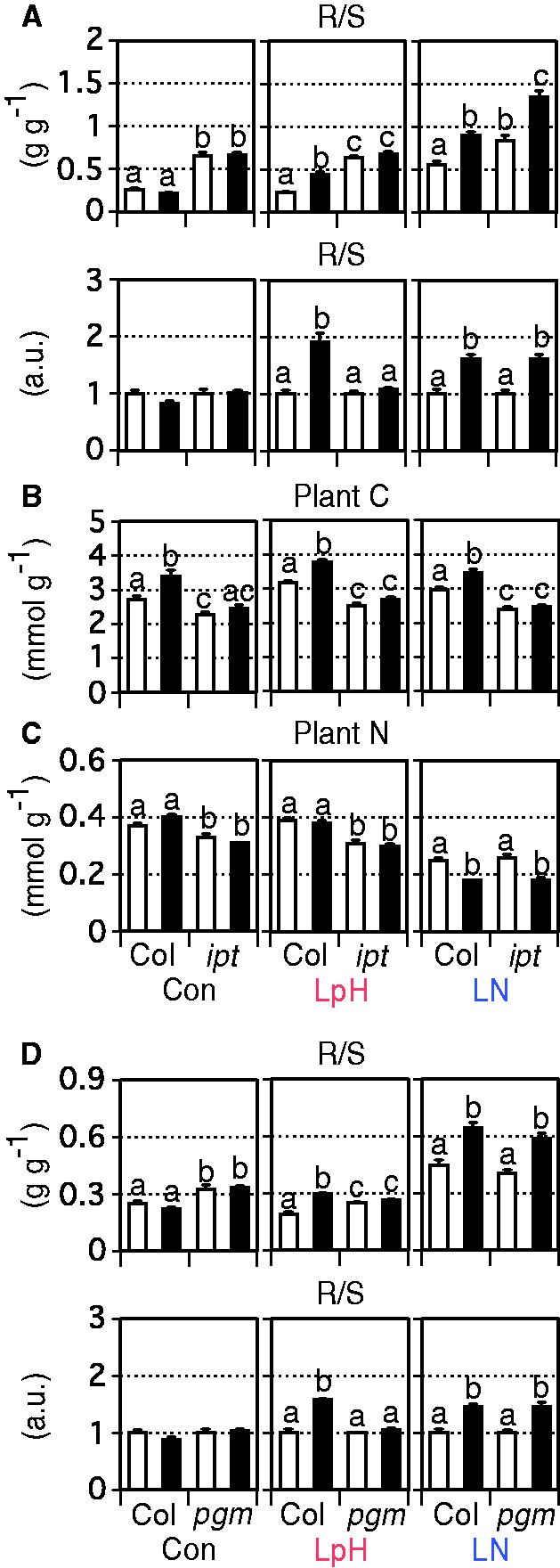
Effects of abiotic stresses and high CO_2_ on (A) the R/S ratio, (B) C concentration and (C) N concentration in 14 d Col-0 and *ipt357* (*ipt*) plants and on (D) the R/S ratio in 14 d Col-0 and *pgm-1* (*pgm*) plants (i.e. 19-day-old plants) (*n* = 6–8). One Col-0 and one mutant plant were grown in each dish. ‘a.u.’ refers to arbitrary units representing values normalized to those obtained at 390 ppmv CO_2_. White and black bars denote ambient (390 ppmv) and high (780 ppmv) CO_2_ conditions, respectively. Con, LpH and LN correspond to control, low-pH and low-N media, respectively. Tukey–Kramer’s multiple comparison test was conducted at a significance level of *P* < 0.05 only when a one-way ANOVA was significant at *P* < 0.05. Different letters denote significant differences. Vertical bars represent the SEM.

To elucidate whether CK is related to PRG, we examined growth of the *ipt357* mutant, a triple mutant for ATP/ADP *ISOPENTENYLTRANSFERASE* genes (*IPT3*, *IPT5* and *IPT7*) (Fig. 2A; Supplementary Fig. S2A–F). This mutant contains extremely low levels of active CKs and has a larger root system than Col-0 (Miyawaki et al. 2006). As expected, R/S ratio absolute values were higher in *ipt357* than in Col-0 under all conditions (Fig. 2A). Under low pH/high CO_2_, the R/S ratio in *ipt357* was not increased by high CO_2_, whereas under low N conditions, responses to high CO_2_ were similar between Col-0 and *ipt357*. Root morphological analysis confirmed that stimulation of LR number under low pH/high CO_2_ was significantly repressed in *ipt357* (Supplementary Fig. S3). These observations indicate that PRG under low pH/high CO_2_ is a CK-related process, and that the mechanisms underlying PRG differ between low pH/high CO_2_ and low N/high CO_2_ conditions.

Under low pH/high CO_2_ and low N/high CO_2_ conditions, PRG might be accompanied by changes in plant C and N status. We therefore measured plant C and N concentrations (Fig. 2B, C). In Col-0, high CO_2_ significantly increased plant C concentrations in all media (Fig. 2B). The maximum C concentration was attained under low pH/high CO_2_. On the other hand, high CO_2_ barely affected plant N concentrations under control and low pH conditions (Fig. 2C). The N concentration was lowest under low N/high CO_2_ conditions. These results suggest that excess C accumulated under low pH/high CO_2_, while N was depleted under low N/high CO_2_. In *ipt357*, C and N concentrations were lower than in Col-0, except for N concentration under low N conditions; in that case, N concentrations were similar between *ipt357* and Col-0. Under high CO_2_, the mutant accumulated less C than Col-0.

We next examined whether the excess soluble C accumulation was related to PRG under low pH/high CO_2_ conditions using *pgm-1*, a *PHOSPHOGLUCOMUTASE* starchless mutant (Fig. 2D; Supplementary Fig. S2A–F). Leaves of *pgm-1* contain high levels of soluble C compounds such as soluble sugars and sugar phosphates even under ambient CO_2_ conditions (Schulze et al. 1991, Gibon et al. 2004, Bläsing et al. 2005). If the excess soluble C accumulation was related to PRG, this mutant should no longer show PRG under low pH/high CO_2_. On the other hand, if N depletion under low N/high CO_2_ was related to PRG, this mutant should show PRG. As expected, *pgm-1* did not exhibit increases in R/S ratios under low pH/high CO_2_ (Fig. 2D), whereas this mutant showed PRG under low N/high CO_2_ similar to that in Col-0. These results indicate that PRG under low pH/high CO_2_ is associated with the excess soluble C accumulation, and that two abiotic stresses induce PRG differently under high CO_2_.

### Effects of low pH and high CO_2_ on concentrations of C compounds in shoots

**Fig. 3:**
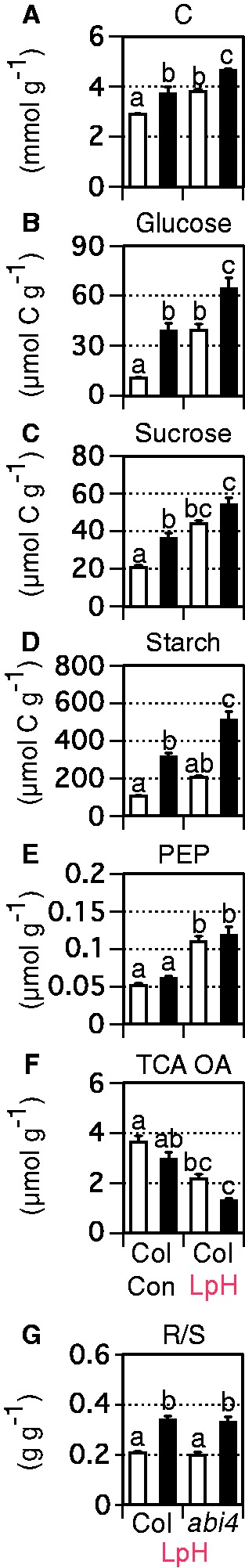
Effects of low pH stress and high CO_2_ on concentrations of (A) C, (B) glucose, (C) sucrose, (D) starch, (E) phosphoenolpyruvate (PEP),and (F) organic acids of the TCA cycle (TCA OA) in 14 d Col-0 shoots (i.e. 19-day-old shoots) and on (G) the R/S ratio of 14 d Col-0 and *abi4-1* (*abi4*) plants (i.e. 19-day-old plants) [*n* = 3 (A), *n* = 4 (B–D), *n* = 5 (E, F), *n* = 8 (G)]. White and black bars denote ambient (390 ppmv) and high (780 ppmv) CO_2_ conditions, respectively. Con and LpH correspond to control and low-pH, respectively. Tukey–Kramer’s multiple comparison test was conducted at a significance level of *P* < 0.05 only when a one-way ANOVA was significant at *P* < 0.05. Different letters denote significant differences. Vertical bars represent the SEM.

To identify the types of C compounds accumulating under low pH/high CO_2_ conditions, we measured concentrations of C, carbohydrates and intermediates of primary metabolic pathways in shoots (Fig. 3). Total C, glucose, sucrose and starch accumulated at the highest levels under low pH/high CO_2_ (Fig. 3A–D). There was no significant difference in fructose concentration between the two CO_2_ regimes under low pH conditions (Supplementary Fig. S4A). Concentrations of phosphoenolpyruvate (PEP; Fig. 3E) and 3-phosphoglycerate (PGA; Supplementary Fig. S4B) were higher under low pH conditions than under control conditions. On the other hand, organic acids in the tricarboxylic acid cycle (TCA OA) were lower under low pH conditions, especially at 780 ppmv CO_2_ (Fig. 3F). These results confirm that excess C accumulates in shoots under low pH/high CO_2_, and identify glucose, sucrose and/or their related C compounds as candidate C-excess signals. The conventional pathway for sugar signaling may thus contribute to PRG. We tested whether PRG was absent in *abi4-1*, a mutant of *ABSCISIC ACID INSENSITIVE4/GIN6/ISI3/SIS5/SUN6* whose locus has been captured by multiple forward genetic screenings for sugar signaling (Rolland et al. 2006). The R/S ratio response of *abi4-1* to CO_2_ under low pH conditions was similar to that of Col-0 (Fig. 3G), however, indicating that PRG under low pH/high CO_2_ is independent of ABI4.

### Effects of low pH and high CO_2_ on concentrations of N compounds in shoots

**Fig. 4:**
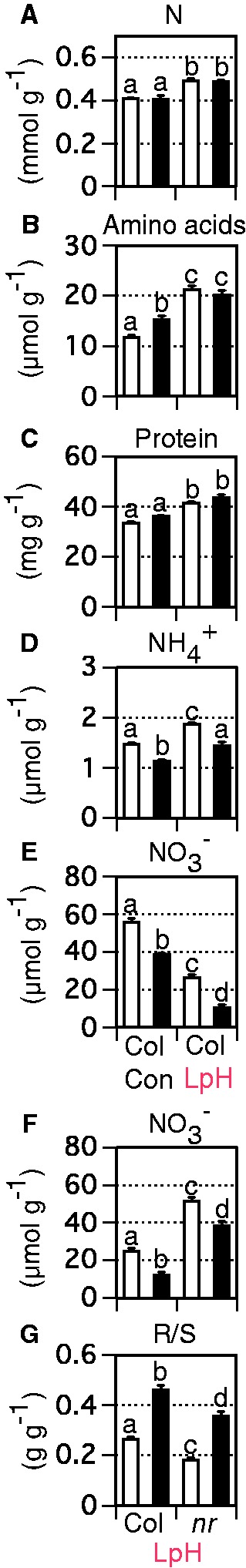
Effects of low pH stress and high CO_2_ on concentrations of (A) N, (B) amino acids, (C) protein, (D) ammonium (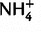) and (E) nitrate (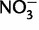) in 14 d Col-0 shoots (i.e. 19-day-old shoots) and on (F) shoot nitrate concentration and (G) the R/S ratio in 14 d Col-0 and *NITRATE REDUCTASE* (*nr*) mutant plants (i.e. 19-day-old plants) [*n* = 3 (A), *n* = 5 (B), *n* = 4 (C–E), *n* = 8 (F, G)]. White and black bars denote ambient (390 ppmv) and high (780 ppmv) CO_2_ conditions, respectively. Con and LpH correspond to control and low-pH, respectively. Tukey–Kramer’s multiple comparison test was conducted at a significance level of *P* < 0.05 only when a one-way ANOVA was significant at *P* < 0.05. Different letters denote significant differences. Vertical bars represent the SEM.

Although we demonstrated that plant N was not depleted under low pH/high CO_2_ conditions (Fig. 2C), the R/S ratio might be systemically regulated by shoot inorganic or organic N (see the Introduction). We thus measured N compound concentrations in shoots (Fig. 4A–E). Concentrations of total N, amino acids, protein and Chl were significantly higher under low pH conditions than under control conditions (Fig. 4A–C; Supplementary Fig. S4C). These results indicate that organic N was never depleted under low pH/high CO_2_. The ammonium concentration was slightly higher under low pH conditions (Fig. 4D), whereas the nitrate concentration was depleted, especially at 780 ppmv (Fig. 4E). It occurred to us that low shoot nitrate levels might result in PRG under low pH/ high CO_2_, as suggested by Scheible et al. (1997). To test this hypothesis, we used *nia1-1nia2-5* (*nr*), a *NITRATE REDUCTASE* double mutant that accumulates more nitrate than Col-0, with nitrate as an N source (Wilkinson and Crawford 1993, Wang et al. 2004). Under low pH conditions, nitrate accumulated in shoots of the NR mutant, with slightly smaller decreases in nitrate concentrations observed in response to high CO_2_ than in Col-0 (Fig. 4F). It should be noted that a similar decrease in nitrate concentrations in response to high CO_2_ was observed in Col-0 shoots under control conditions (Fig. 4E, F). The NR mutant had a lower R/S ratio than Col-0 at 390 ppmv, confirming the observations of Scheible et al. (1997) (Fig. 4G). Nevertheless, the NR mutant showed PRG similar to that of Col-0 (Fig. 4G). These results suggest that PRG under low pH/high CO_2_ is independent of nitrate depletion.

### Effects of sugar application on PRG

**Fig. 5:**
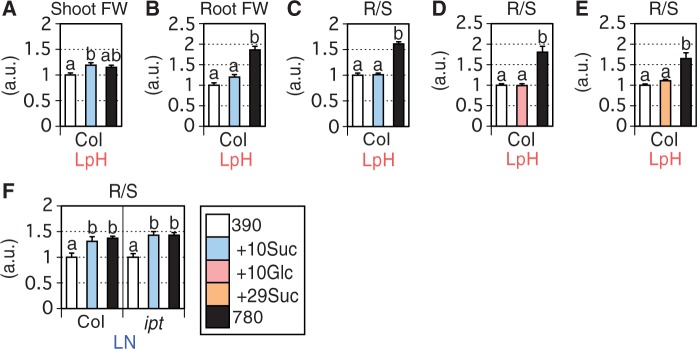
Effects of low pH stress, high CO_2_ and 10 mM sucrose application on (A) shoot FW, (B) root FW and (C) the R/S ratio in 14 d Col-0 plants (i.e. 19-day-old plants), effects of low pH stress, high CO_2_ and 10 mM glucose application on (D) the R/S ratio in 14 d Col-0 plants (i.e. 19-day-old plants), effects of low pH stress, high CO_2_ and 1% (w/v) (∼29 mM) sucrose application on (E) the R/S ratio in 14 d Col-0 plants (i.e. 19-day-old plants) and effects of low N stress, high CO_2_ and 10 mM sucrose application on (F) the R/S ratio in 14 d Col-0 and *ipt357* plants (i.e. 19-day-old plants) (*n* = 6–8). 10Suc, 10Glc and 29Suc denote 10 mM sucrose, 10 mM glucose and 29 mM sucrose, respectively. White and black bars denote ambient (390 ppmv) and high (780 ppmv) CO_2_ conditions, respectively. LpH and LN correspond to low-pH and low-N media, respectively. Tukey–Kramer’s multiple comparison test was conducted at a significance level of *P* < 0.05 only when a one-way ANOVA was significant at *P* < 0.05. Different letters denote significant differences. Vertical bars represent the SEM.

Under low pH/high CO_2_ conditions, excess C accumulation in shoots may increase C availability in roots, leading to PRG. We therefore hypothesized that exogenous sugar application to roots under ambient CO_2_ may mimic the effects of high CO_2_ on PRG. Based on sucrose concentration measurements (Fig. 3C), sucrose at physiological concentration was added to the low pH medium. The sugar applications increased growth of shoots and roots equally, and thus did not cause PRG (Fig. 5A–C). Applications of 10 mM glucose or 1% (w/v) sucrose (∼29 mM) also did not mimic the effects of high CO_2_ on PRG (Fig. 5D, E). These results suggest that PRG under low pH conditions is not related to increases in root C availability.

On the other hand, under low N conditions, 10 mM sucrose applications at 390 ppmv induced PRG in both Col-0 and *ipt357* (Fig. 5F). This result further confirmed that the underlying mechanisms for PRG differ between low pH/high CO_2_ and low N/high CO_2_ conditions.

### Relationship between auxin/CK and PRG

**Fig. 6:**
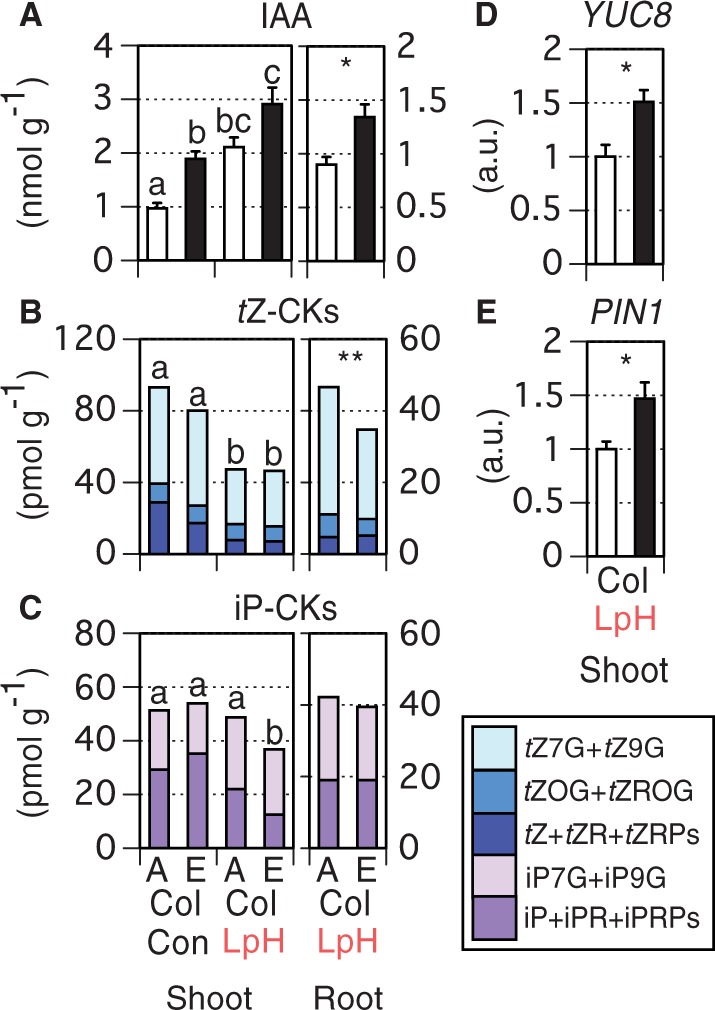
Effects of low pH stress and high CO_2_ on concentrations of (A) IAA, (B) *t*Z-type CKs and (C) iP-type CKs in 14 d Col-0 shoots (*n* = 4) and roots (*n* = 3) (i.e. 19-day-old shoots and roots) and on transcript levels of (D) *YUC8* and (E) *PIN1* in 14 d Col-0 shoots (i.e. 19-day-old shoots) (*n* = 3). White and black bars denote ambient (390 ppmv) and high (780 ppmv) CO_2_ conditions, respectively. ‘A’ and ‘E’ refer to ‘ambient CO_2_ (390 ppmv)’ and ‘high CO_2_ (780 ppmv)’, respectively. Con and LpH correspond to control and low pH, respectively. Tukey–Kramer’s multiple comparison test was conducted at a significance level of *P* < 0.05 only when a one-way ANOVA was significant at *P* < 0.05. Different letters denote significant differences. Student’s *t*-test was conducted (*P < 0.05; **P < 0.01; ***P < 0.001). Vertical bars represent the SEM.

Our results demonstrate that PRG under low pH/high CO_2_ is accompanied by an enhancement in LR number and is closely related to CK activity and C accumulation. These findings suggest that auxin is a key player in PRG: auxin enhances LR formation, an effect that can be partly antagonized by CKs, and metabolizable sugars can induce LR and auxin biosynthesis (Laplaze et al. 2007, Fukaki and Tasaka 2009, Mishra et al. 2009, Lilley et al. 2012, Sairanen et al. 2012). In addition, shoot-derived auxin can function as a long-distance signal to enhance LR growth and may decrease root CK contents (Reed et al. 1998, Bhalerao et al. 2002, Nordström et al. 2004, Takei et al. 2004, Lilley et al. 2012). We therefore measured concentrations of IAA and CKs in shoots and roots (Fig. 6). In shoots, IAA concentrations (Fig. 6A) were positively correlated with concentrations of C (*r* = 0.997, *P* = 0.003; Fig. 3A), glucose (*r* = 0.995, *P* = 0.005; Fig. 3B) and sucrose (*r* = 0.990, *P* = 0.010; Fig. 3C). In addition, root IAA concentrations were higher at 780 ppmv than at 390 ppmv under low pH conditions. On the other hand, shoot *t*Z-type CK concentrations were much lower under low pH than under control conditions (Fig. 6B). Root *t*Z-type CK concentrations were significantly lower under low pH/high CO_2_, and shoot iP-type CK concentrations were lowest under low pH/high CO_2_ (Fig. 6C). In roots under low pH conditions, no significant difference in iP-type CKs was observed between the two CO_2_ regimes. Furthermore, we observed a significant increment in mRNA of *PIN1*, a polar auxin efflux transporter gene, and *YUCCA8*, an auxin biosynthetic gene, under low pH/high CO_2_ conditions (Fig. 6D, E). These results support the notion that excess C accumulation in shoots could induce PRG via the actions of IAA and CK under low pH/high CO_2_ conditions.

## Discussion

Although several meta-analyses have suggested that high CO_2_ can cause PRG, observed responses are often small or negligible (e.g. Rogers et al. 1996). Poorter and Nagel (2000) found no significant effect of high CO_2_ on PRG. In addition, in preliminary experiments using an MGRL-based medium (Fujiwara et al. 1992) or a half-strength Murashige and Skoog (MS) medium (Murashige and Skoog 1962), we found that high CO_2_ stimulated growth of *A. thaliana* shoots and roots almost equally (data not shown). Based on meta-analyses reported in the literature, we hypothesized that abiotic stress might be a prerequisite for PRG. In fact, we found that low pH and low N stresses induced PRG under high CO_2_ conditions (Fig. 1). Under low pH/high CO_2_, large quantities of C accumulated in plants (Fig. 2B), whereas N was severely depleted under low N/high CO_2_ (Fig. 2C). These two stresses gave rise to similar phenomena because they both induced increases in LR number (Fig. 1J) and C/N ratios in plants (Fig. 2B, C); however, phenotypic analyses of *ipt357* and *pgm-1* and sugar application experiments clarified that these stresses caused PRG via distinct mechanisms (Figs. 2A, D, 5C, D). ‘C excess’ and ‘N depletion’ regulate R/S ratios differently under low pH and low N stresses, respectively. In this study, we focused on low pH/high CO_2_ because PRG under these conditions is a high CO_2_-specific (Fig. 5C) and CK-related phenomenon (Fig. 2A; Supplementary Fig. S2). A metabolomic analysis showed accumulation of soluble sugars under low pH/high CO_2_ (Fig. 3B, C). The obtained results combined with literature information suggest auxin as a missing link. Plant hormone profiling and gene expression analyses indicated that excess C accumulation can enhance LR growth via dual actions of increased auxin and decreased CKs (Fig. 6). A possible model for PRG under low pH/high CO_2_ is shown in Supplementary Fig. S6.

### How does excess C accumulation increase IAA concentrations?

Recent findings indicate that sugar applications can induce auxin biosynthesis via a PIF transcription factor-related mechanism (Lilley et al. 2012, Sairanen et al. 2012). This induction is associated with increased expression of several IAA biosynthetic genes, including *YUCCA8* and *YUCCA9*. Consequently, we measured transcript levels of *YUCCA* genes in shoots grown at low pH (Fig. 6D; Supplementary Fig. S5). Only *YUCCA8* was significantly induced at 780 ppmv, implying that this gene might be responsible for the IAA accumulation observed under low pH/high CO_2_. In another study, however, a double knockout of *YUCCA8* and *YUCCA9* had only minor effects on sugar-dependent IAA accumulation (Sairanen et al. 2012). A tissue-specific expression analysis in mature plants has revealed that *YUCCA8* is expressed in sink leaves more strongly than in source leaves (Hentrich et al. 2013). Sink leaves may sense imported sugars or their derivatives, and function as a primary IAA source. High temperature treatment can induce *YUCCA8* and *PIF4* concomitantly, which is followed by IAA accumulations in *A. thaliana* seedlings (Sun et al. 2012). PIF4 binds to the promoter region of *YUCCA8*, and disruption of *PIF4* suppresses the high temperature-dependent IAA accumulation. In this case, PIF4 acts as a positive regulator. On the other hand, *PIF4* transcript levels are not changed by sugar treatments (Lilley et al. 2012). We likewise did not observe *PIF4* induction in response to high CO_2_ (Supplementary Fig. S5). Sairanen et al. (2012) observed that sugar-dependent IAA biosynthesis is suppressed in a *PIF5*-overexpressor line, but enhanced in a *pif1pif3pif4pif5* quadruple mutant, suggesting that PIFs are negative regulators. PIFs and YUCCA8 may be related to IAA accumulation under low pH/high CO_2_ conditions.

### How is C excess information in shoots transmitted to roots?

Given that shoot-derived IAA could be a C-excess systemic signal, there are two pathways for shoot to root transport of auxin: fast, non-directional transport in the phloem, and slow, directional, so-called polar auxin transport via PIN proteins in various tissues (Friml and Palme 2002). Lilley et al. (2012) have demonstrated that IAA transport from shoots to roots is enhanced under high sugar or high light conditions in *A. thaliana* seedlings. A shoot to root auxin transport via PIN1 plays a significant role in light enhancement of root growth (Sassi et al. 2012). Sugar applications increase *PIN1* and *PIN7* transcript levels (Mishra et al. 2009, Lilley et al. 2012). We also observed a significant increment in *PIN1* mRNA under low pH/high CO_2_ conditions (Fig. 6E). *PIN1* transcript levels and functional localization of PIN1 proteins on the plasma membrane are reduced by CK action, while *PIN1* expression is induced by auxin application (Laplaze et al. 2007, Růžička et al. 2009, Marhavý et al. 2011). The increased IAA and decreased CKs might enhance shoot to root transport of IAA via PIN1 (Supplementary Fig. S6). In *aux1*, a mutant of an auxin influx carrier, IAA content is lower in root tips, but higher in young leaves compared with Col-0 (Swarup et al. 2001, Marchant et al. 2002). Li et al. (2011) have suggested that shoot-supplied ammonium inhibits LR growth by suppressing *AUX1* expression. These results imply that AUX1 may play a role in shoot to root IAA transport, although its transcript levels were unchanged under high CO_2_ (Supplementary Fig. S5).

Sugar application can induce auxin biosynthesis in both shoots and roots (Sairanen et al. 2012). Under low pH/high CO_2_, enhanced sugar transport into roots may therefore increase IAA biosynthesis and accumulation in roots, inducing PRG (Fig. 6A). In our study, however, addition of sugar to the medium did not cause PRG (Fig. 5C). No significant difference in root C concentrations was observed between the two CO_2_ regimes under low pH conditions (3.33 ± 0.12 mmol C g FW^−1^ at 390 ppmv, 3.17 ± 0.12 mmol C g FW^−1^ at 780 ppmv). These observations suggest that increased root C availability does not trigger PRG under low pH conditions. It is reasonable to state that shoot-derived signals such as auxin may stimulate root growth, which in turn increases C demand in roots. A sugar-dependent stimulation of LR growth has been reported to be weak in several mutants related to auxin signaling (Mishra et al. 2009).

### How is PRG suppressed in a CK-deficient mutant?

CKs can suppress LR growth by at least two pathways: one is a direct pathway that inhibits the LR initiation process in LR founder cells, and the other is mediated via suppression of polar auxin transport (Laplaze et al. 2007, Růžička et al. 2009, Marhavý et al. 2011). Consequently, only a root-specific reduction of CKs is sufficient to induce root growth (Werner et al. 2010). In *ipt357*, PRG in response to high CO_2_ was suppressed, whereas shoot growth CO_2_ responses were similar between Col-0 and *ipt357* (Fig. 2A; Supplementary Fig. S2A–F). Our hormone analysis suggested that decreased levels of *t*Z-type CKs observed in roots may be related to PRG in Col-0. It is possible that in *ipt357*, a severe CK-deficient mutant, CKs could not decrease in response to high CO_2_, which led to the suppression of PRG. Soluble sugars and starch are significantly decreased in CK-deficient tobacco (*35S:CKX1*, *35S:CKX2*) leaves (Werner et al. 2008). In addition, *ipt357* plants did not accumulate C under low pH/high CO_2_ conditions (Fig. 2B). In *ipt357*, an enlarged root system (i.e. a strong sink) might consume shoot-derived sugar to avoid excess C accumulation in shoots. Alternatively, a small shoot might limit photosynthetic C acquisition in this mutant. This decreased C accumulation may not induce auxin biosynthesis in shoots, preventing PRG induction. In roots of a CK-deficient transgenic line, *PIN1* expression has been found to be up-regulated (Růžička et al. 2009). It is therefore possible that fluxes of polar auxin transport for PRG are saturated in *ipt357*.

### How does N depletion cause PRG?

Under low N/high CO_2_ conditions, plant growth stimulated by high CO_2_ depleted N in plants and media, possibly causing PRG (Figs. 1, 2C). This PRG was accompanied by increases in LR numbers (Fig. 1J). In soybean and *A. thaliana*, low N conditions increase root IAA concentrations (Caba et al. 2000, Walch-Liu et al. 2006). In contrast, shoot IAA concentrations are not increased under these conditions (Walch-Liu et al. 2006). Krapp et al. (2011) found that complete N starvation induces accumulation of soluble sugars in roots compared with shoots. In our study, we observed that sugar application to roots caused PRG under low N/ambient CO_2_ conditions (Fig. 5D). Consequently, enhanced sugar supply from shoots to roots may induce IAA biosynthesis in roots (Sairanen et al. 2012). Decreased amino acid content in roots may enhance LR growth under low N conditions (Gifford et al. 2008, Pellny et al. 2008). Gifford et al. (2008) have revealed that root amino acids control LR growth via an auxin response factor 8 (ARF8)/miR167 circuit. It appears that auxin is also a key player in PRG under low N conditions.

### Challenges for the future

In the present study, we obtained a comprehensive view of metabolic and hormonal changes in 14 d plants grown under low pH/high CO_2_ conditions. However, it is difficult to determine whether these changes are causes for PRG induction or consequences of PRG and its relevant physiological alteration. A detailed time-course analysis which focuses on soluble C compounds and plant hormones would improve the accuracy of the proposed model (Supplementary Fig. S6). The link between high C status and auxin action has been analyzed using very young seedlings with short sugar treatment (Mishra et al. 2009, Lilley et al. 2012, Sairanen et al. 2012). Therefore, it is a challenging issue to reveal whether the mechanisms thus obtained could also act in mature plants exposed to high CO_2_ for a prolonged period. Mutants related to auxin biosynthesis, transport and signaling should also be useful for unraveling auxin’s role in PRG induction. A study like this can build a bridge between molecular work and field research.

### Conclusions

Under low pH/high CO_2_ conditions, excess C is accumulated in shoots, which in turn might induce IAA biosynthesis in shoots and IAA transport into roots (Supplementary Fig. S6). IAA in roots might promote LR growth directly and/or indirectly via suppression of CK action. Under low N/high CO_2_, the high CO_2_ levels would stimulate plant growth and rapidly deplete N in plants and media, causing PRG. Multiple lines of evidence obtained in the present study indicate that two distinct systems trigger PRG under low pH and low N stress conditions at high CO_2_.

## Materials and Methods

### Plant materials and growth conditions

*Arabidopsis thaliana* (L.) Heynh. ecotype Col-0 and mutants *ipt357*, a triple mutant for ATP/ADP *ISOPENTENYLTRANSFERASE* genes (*IPT3*, *IPT5* and *IPT7*) (Miyawaki et al. 2006), *pgm-1*, a *PHOSPHOGLUCOMUTASE* starchless mutant (Schulze et al. 1991), *abi4-1*, a mutant of *ABSCISIC ACID INSENSITIVE4* (Finkelstein 1994), and *nia1-1nia2-5* (*nr*), a *NITRATE REDUCTASE* double mutant (Wilkinson and Crawford 1993), were used in our experiments. Seeds of *pgm-1*, *abi4-1* and *nia1-1nia2-5* were purchased from the Arabidopsis Biological Resource Center, and seeds of *ipt357* were generous gifts from Professor T. Kakimoto (Osaka University, Japan). After surface sterilization, 150 seeds were sown on plastic Petri dishes (length 140 mm, width 100 mm, depth 20 mm; Eiken Kagaku) containing 50 ml of an N-modified one-sixth-strength MS medium with 10 mM KNO_3_, 5 mM NH_4_(SO_4_)_2_, 1% (w/v) sucrose and 0.8% (w/v) agar (Hachiya et al. 2012). After sowing, the plates were kept at 4°C in the dark for 2 d. Plants were germinated in a high-performance CO_2_-controlled growth chamber (LPH-0.5P-SH; Nippon Medical & Chemical Instrument) at 390 ppmv under a photosynthetic photon flux density of 100–130 µmol m^−2 ^s^−1^ (10 h light/14 h dark cycle) at 23°C and 60% relative humidity. Five-day-old seedlings were selected for size uniformity and transferred to control, low-pH or low-N media. These media included one-sixth-strength MS macro- and micronutrient salts (except for N) containing 2 mM KCl, 0.85 mM MES and 1% (w/v) agar. Control and low-pH media included 2 mM N (0.5 mM NH_4_NO_3_ and 1 mM NH_4_Cl), whereas the low-N medium contained only 0.4 mM N (0.1 mM NH_4_NO_3_ and 0.2 mM NH_4_Cl). The pH values of solidified media were adjusted to 6.1 (control), 4.9 (low pH) or 6.1 (low N) with HCl or KOH. Note that these media contained no sucrose. Two plants per dish were grown in a horizontal position on plastic dishes (diameter 90 mm, depth 20 mm; Iwaki) containing 30 ml of media for 2 weeks, or, alternatively, three plants per dish were grown in a vertical position on plastic dishes (length 140 mm, width 100 mm, depth 20 mm; Eiken Kagaku) containing 50 ml of media for 10 d. Further details are given in the Results and the figure legends.

### Growth analysis and root morphological analysis

For the growth analysis, 14 d plants after the transfer were harvested and scanned at 300 dpi resolution for measurement of rosette diameter. Plants were then divided into leaves, roots and residual parts, each of which was weighed on a precision balance. Leaves (>1 mm long leaf blades) were scanned at 300 dpi for measurements of leaf area and leaf number. Rosette diameter and leaf area were measured using ImageJ software (ver. 1.43).

For the root morphological analysis, the outline of roots of 10 d plants after the transfer were drawn on the plastic dish with a fine-tipped marker. The traced drawings were scanned at 300 dpi for measurements of length and number of primary roots and LRs (>0.5 mm). These analyses were also conducted using ImageJ.

### Determination of nitrate, N and C concentrations

For the determination of nitrate concentrations, one shoot from each dish was sampled 3.5–5.5 h after daybreak. The samples were immediately weighed, washed with deionized water, dried at 80°C for at least 3 d and kept in a sealed container with silica gel before use. Nitrate concentrations were determined according to Hachiya et al. (2010). For determinations of C and N concentrations, two shoots per dish were sampled 3.5–5.5 h after daybreak, or, alternatively, one plant per dish was sampled after the growth analysis. Samples were weighed, washed and dried as described above, and ground into powder. C and N concentrations were determined with a CN analyzer (Vario EL; Elementar Analysensysteme GmbH) according to Hachiya and Noguchi (2008).

### Determination of ammonium and protein concentrations

To determine ammonium and protein concentrations, two shoots per dish were sampled 3.5–5.5 h after daybreak, washed with de-ionized water, frozen in liquid N_2_ and stored at −80°C. Ammonium was extracted according to Schneidereit et al. (2006) and Bräutigam et al. (2007) with slight modifications. Frozen shoots were ground with a Multi-Beads Shocker (Yasui Kikai) using a metal cone (MC-0212; Yasui Kikai) at 2,000 r.p.m. for 10 s, and 0.1 N HCl was then added to the frozen powder. The extract was kept on ice for 30 min, and then centrifuged at 10,000×*g* at 4°C for 10 min. The supernatants were purified by acid-washed activated charcoal (035-18081; Wako) and chloroform to eliminate interfering compounds. Ammonium in the extract was determined using an ammonia test kit (Wako) according to the manufacturer’s instructions. Total protein was extracted and determined according to Hachiya et al. (2007).

### Determinations of glucose, sucrose, fructose and starch concentrations

For the determination of carbohydrates, two shoots per dish were sampled 3.5–5.5 h after daybreak, weighed, frozen in liquid N_2_ and stored at −80°C. Frozen shoots were ground with a Multi-Beads Shocker (Yasui Kikai) using a metal cone (Yasui Kikai) at 2,000 r.p.m. for 10 s, and ice-cold 80% (v/v) ethanol was then added to the frozen powder. The mixtures were incubated at 80°C for 10 min, and centrifuged at 13,000×*g* at 4°C for 10 min. The extraction process after precipitation was repeated three times to improve the recovery rate. Glucose, sucrose and fructose in the extract and starch in the precipitate were determined according to Hachiya and Noguchi (2008).

### Determination of primary metabolites using capillary electrophoresis-mass spectrometry (CE-MS)

Extraction and determination of primary metabolites was conducted according to Sato et al. (2004) and Sato and Yanagisawa (2010). Two shoots per dish were sampled 3.5–5.5 h after daybreak, weighed, frozen in liquid N_2_ and stored at −80°C. Frozen shoots were ground with a Multi-Beads Shocker (Yasui Kikai) using a metal cone (Yasui Kikai) at 2,000 r.p.m. for 10 s, and 200 µl of ice-cold 80% (v/v) methanol was then added to the frozen powder. After vigorous agitation, an equivalent volume of an internal standard solution containing 200 µM PIPES and l-methionine sulfone was added to the extract and mixed for 5 min at 4°C. The mixture was centrifuged at 5,000×*g* at 4°C for 1 min. The supernatant was poured onto a 3 kDa cut-off membrane (PALL) and centrifuged at 15,000×*g* for 15 min at 4°C. Filtered samples were freeze-dried using a Christ freeze dryer (Kubota) and stored at −80°C. Samples were dissolved in MilliQ water and analyzed on an Agilent CE capillary electrophoresis system with a built-in diode array detector, an Agilent 1100 series MSD mass spectrometer, an Agilent 1100 series isocratic HPLC pump, a G1603A Agilent CE-MS adapter kit and a G1607A Agilent CEESI-MS sprayer kit (Agilent Technologies). System control, data acquisition and MSD data evaluation were performed using G2201AA Agilent ChemStation software for CE-MSD. Metabolite levels obtained were corrected against internal standards. In this study, TCA OA concentration was calculated as the sum of citrate, fumarate, malate, oxaloacetate, 2-oxoglutarate and succinate concentrations. The oxaloacetate concentration was estimated based on glutamate oxaloacetate transaminase equilibrium (Shen et al. 2006). The amino acid concentration corresponded to the sum of alanine, arginine, asparagine, aspartate, glutamine, glutamate, glycine, histidine, isoleucine, leucine, methionine, phenylalanine, serine, threonine and tryptophan concentrations. We were not able to detect significant concentrations of cysteine, lysine, proline, tyrosine and valine.

### Determination of plant hormones using ultra-perfomance liquid chromatography-electrospray ionization-tandem quadrupole mass spectrometry

Two shoots from one dish and 10 roots from five dishes were sampled 3.5–5.5 h after daybreak, weighed, frozen in liquid N_2_ and stored at −80°C. Extraction and determination of primary metabolites was conducted according to Kojima et al. (2009). *t*Z, *t*ZR, *t*ZRPs, *t*ZOG, *t*ZROG, *t*Z7G and *t*Z9G were determined as *t*Z-type CKs, and iP, iPR, iPRPs, iP7G and iP9G were determined as iP-type CKs.

### Extraction of total RNA, reverse transcription and real-time PCR

Two shoots per dish were sampled 3.5–5.5 h after daybreak, frozen with liquid N_2_ and stored at −80°C. Total RNA was extracted using TRIzol reagent (Life Technologies) according to the manufacturer’s instructions and digested twice with DNase using a TURBO DNA-free kit (Life Technologies). There was no significant difference in RNA concentrations on a FW basis between samples grown at 390 and 780 ppmv under low pH conditions. Reverse transcription was performed with a High Capacity RNA-to-cDNA kit (Life Technologies) according to the manufacturer’s instructions.

Transcript levels were measured using an ABI Prism 7300 sequence detection system (Applied Biosystems). cDNA (1 µl) was amplified in the presence of 12.5 µl of Power SYBR Green PCR Master Mix (Applied Biosystems), 0.5 µl of specific primers (0.2 µM final concentration) and 10.5 µl of sterilized water. PCR conditions were 50°C for 2 min, 95°C for 10 min, and 40 cycles of 95°C for 15 s followed by 60°C for 1 min. We chose *18S rRNA* as the internal standard from three candidates (*18S rRNA*, *ACTIN3* and *EF1α*) because there was no significant difference in C*t* values for *18S rRNA* between samples grown at 390 and 780 ppmv under low pH conditions. We could not find suitable internal control genes under control conditions. Mean transcript levels are shown according to Hachiya et al. (2012) in Fig. 6D, E and Supplementary Fig. S5. Primer sequences used for the experiments are shown in Supplementary Table S1.

### Statistical analysis

All statistical analyses were conducted using the R software package (ver. 2.15.3). Details of analyses are given in the Results and in the figure legends.

## Supplementary data

Supplementary data are available at PCP online.

## Funding

This work was supported by a Grand-in-Aid for Scientific Research on Innovative Areas [No. 21114007] from the Ministry of Education, Culture, Sports, Science and Technology of Japan (MEXT).

## Disclosures

The authors have no conflicts of interest to declare.

## Supplementary Material

Supplementary Data

## Abbreviations

ABI: abscisic acid-insensitive
ANOVA: analysis of variance
ARF: auxin response factor
CE-MS: capillary electrophoresis-mass spectrometry
CK: cytokinin
CKX: cytokinin oxidase
GIN: glucose insensitive
iP: *N*^6^-(Δ^2^-isopentenyl) adenine
IPT: isopentenyltransferase
ISI: impaired sucrose-induction
LR: lateral root
NR: nitrate reductase
PEP: phosphoenolpyruvate
PGA: 3-phosphoglycerate
PGM: phosphoglucomutase
PIF: PHYTOCHROME-INTERACTING FACTOR
PIN: PIN-FORMED
ppmv: parts per million by volume
PRG: preferential root growth
R/S ratio: root to shoot ratio by weight
SIS: sugar-insensitive
SUN: sucrose-uncoupled
TCA OA: organic acids in the tricarboxylic acid cycle
*t*Z: *trans*-zeatin
