# Supplementary material for: High CO2 Triggers Preferential Root Growth of Arabidopsis thaliana Via Two Distinct Systems Under Low pH and Low N Stresses
Source: Plant Cell Physiol. 2014 Jan 30;55(2):269–80. doi: 10.1093/pcp/pcu001 (PMC3913443; doi:10.1093/pcp/pcu001)
Supplement: Supplementary Data [file supp_55_2_269__index.html]

High CO2 triggers preferential root growth of Arabidopsis thaliana via two distinct systems at low pH and low N stresses — High CO2 Triggers Preferential Root Growth of Arabidopsis thaliana Via Two Distinct Systems Under Low pH and Low N Stresses — High CO2 Triggers Preferential Root Growth of Arabidopsis thaliana Via Two Distinct Systems Under Low pH and Low N Stresses — Supplementary Data 

# High CO2 Triggers Preferential Root Growth of *Arabidopsis thaliana* Via Two Distinct Systems Under Low pH and Low N Stresses

## Supplementary Data

files

**Files in this Data Supplement:**

- Supplementary Data - pdf file
