# Supplementary material for: High CO2 Triggers Preferential Root Growth of Arabidopsis thaliana Via Two Distinct Systems Under Low pH and Low N Stresses
Source: Plant Cell Physiol. 2014 Jan 30;55(2):269–80. doi: 10.1093/pcp/pcu001 (PMC3913443; doi:10.1093/pcp/pcu001)
Supplement: Supplementary Data [file supp_pcu001_pcp-2013-e-00437-File008.pdf]

Supplementary Data

Figure S1 Effects of low pH stress and elevated CO_2 on shoot FW (A), root FW (B) and R/S ratio (C) of 21-d plants in Col-0 (n = 5).

Figure S2 Effects of low pH stress, elevated CO_2 and sucrose application on leaf FW (A), residual aboveground FW (B), leaf number (C), leaf area (D), rosette diameter (E) and root FW (F) of 14-d plants in Col-0, *ipt357*, *pgm-1* and *nia1-1/nia2-5* (*nr*) (n = 6-8).

Figure S3 Effects of low pH stress and elevated CO_2 on primary root length (A), total LR length (B), total LR number (C) and mean LR length (D) of 10-d plants in Col-0 and *ipt357* (n = 11-14). Representative traces of roots are shown (E). Scale bar represents 1 cm.

Figure S4 Effects of low pH stress and high CO_2 on concentrations of fructose (A), 3-PGA (B) and Chlorophyll (C) of 14-d shoots in Col-0 (n = 4 in A, n = 5 in B, n = 3 in C).

Figure S5 Effects of low pH stress and elevated CO_2 on transcript levels of genes related to IAA biosynthesis and transport of 14-d shoots in Col-0 (n = 3 in A, n = 5 in B).

Figure S6 Model of PRG under low pH/high CO_2 conditions. Low pH inhibits shoot

growth. Both low pH and high CO_2 enhance C and sugar accumulation in shoots. Excess C accumulation induces IAA biosynthesis and transport into roots. Transported IAA promotes LR growth directly and/or indirectly via suppression of CK signaling.

FIGURE S1

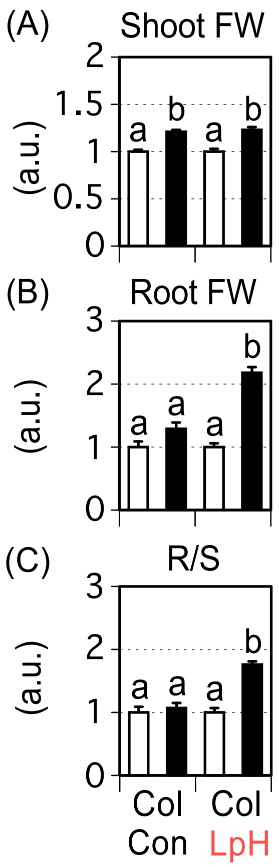

(A)
Shoot FW
(a.u.)
2
1.5
1
0.5
0
a
b
a
b
(B)
Root FW
(a.u.)
3
2
1
0
a
a
a
b
(C)
R/S
(a.u.)
3
2
1
0
a
a
a
b
Col
Con
Col
LpH

FIGURE S2

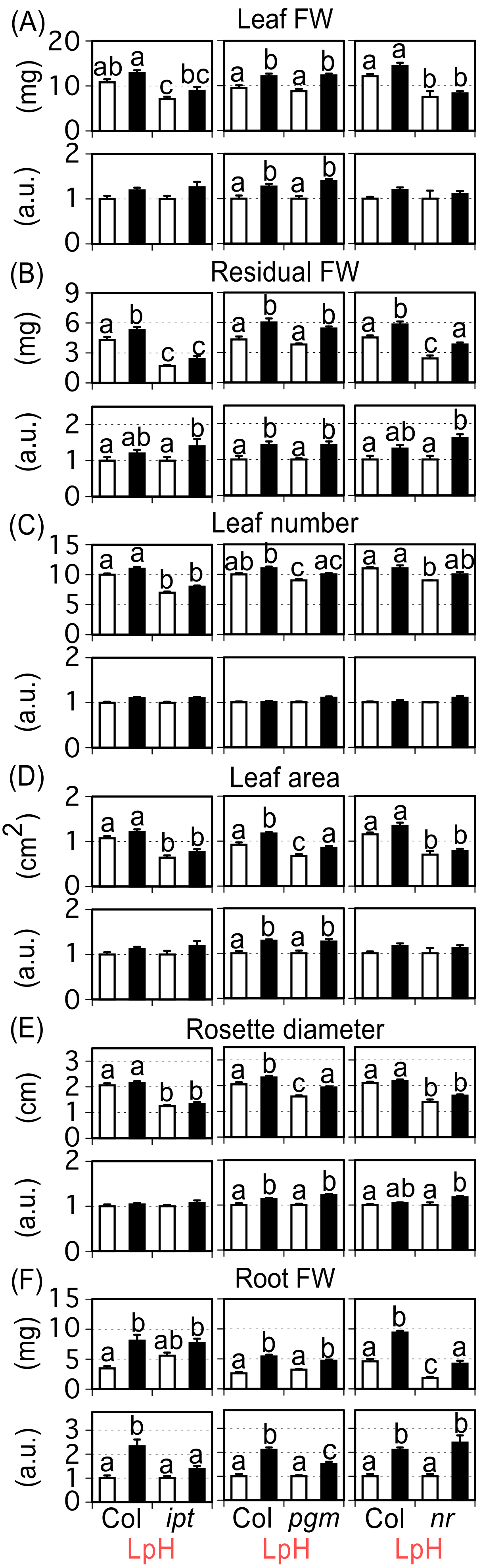

(A)
Leaf FW
(mg)
(a.u.)
(B)
Residual FW
(mg)
(a.u.)
(C)
Leaf number
(a.u.)
(D)
Leaf area
(cm2)
(a.u.)
(E)
Rosette diameter
(cm)
(a.u.)
(F)
Root FW
(mg)
(a.u.)
Col ipt
LpH
Col pgm
LpH
Col nr
LpH

FIGURE S3

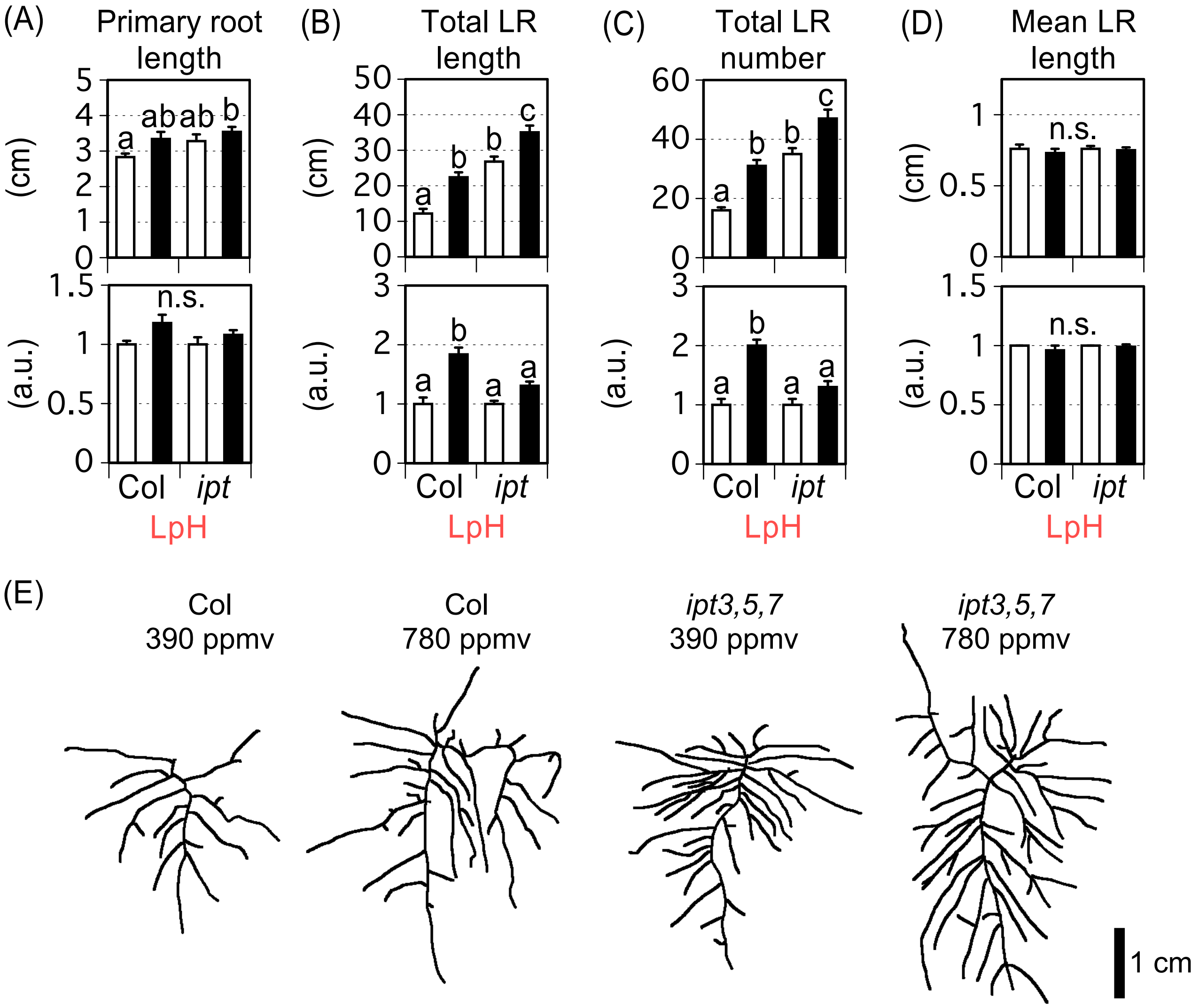

(A)
Primary root length
(cm)
(a.u.)
a
ab
ab
b
n.s.
Col
ipt
LpH
(B)
Total LR length
(cm)
(a.u.)
a
b
b
c
a
b
a
a
Col
ipt
LpH
(C)
Total LR number
a
b
b
c
a
b
a
a
Col
ipt
LpH
(D)
Mean LR length
(cm)
(a.u.)
n.s.
n.s.
Col
ipt
LpH
(E)
Col
390 ppmv
Col
780 ppmv
ipt3,5,7
390 ppmv
ipt3,5,7
780 ppmv
1 cm

FIGURE S4

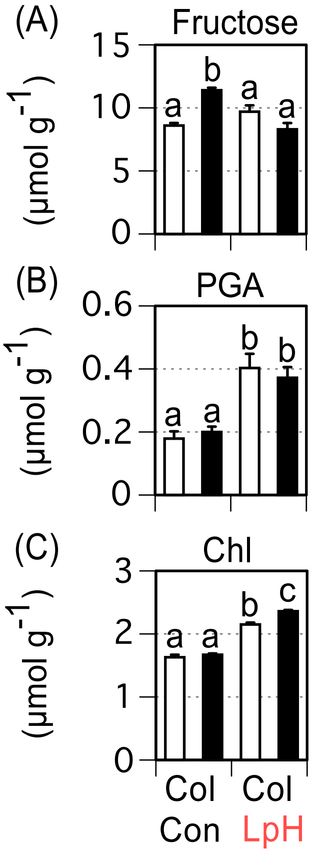

(A)
Fructose
(µmol g-1)
15
10
5
0
a
b
a
a
(B)
PGA
(µmol g-1)
0.6
0.4
0.2
0
a
a
b
b
(C)
Chl
(µmol g-1)
3
2
1
0
a
a
b
c
Col
Con
Col
LpH

FIGURE S5

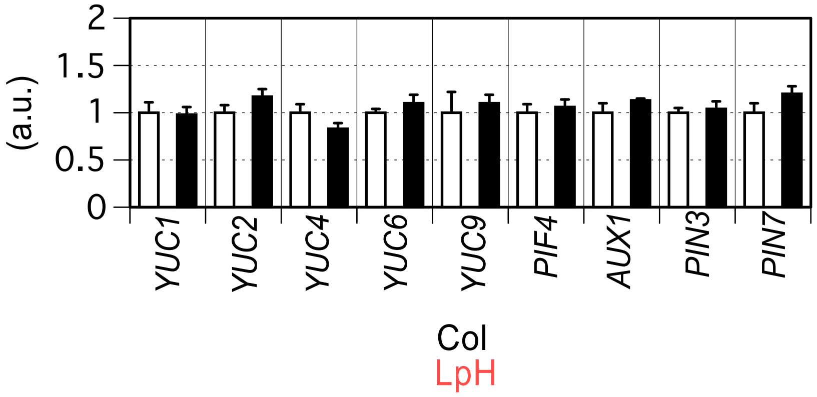
2
1.5
1
0.5
0
(a.u.)
YUC1
YUC2
YUC4
YUC6
YUC9
PIF4
AUX1
PIN3
PIN7
Col
LpH

FIGURE S6

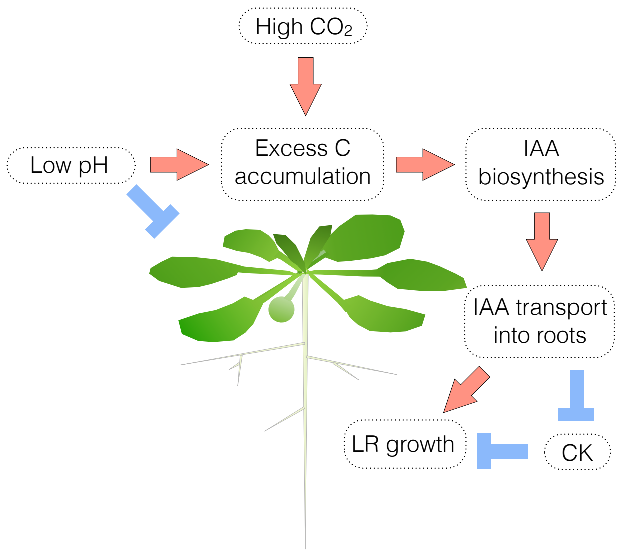
High CO_2
Low pH
Excess C accumulation
IAA biosynthesis
IAA transport into roots
LR growth
CK

Table S1. Primer seqences for Q-PCR analysis in Supplementary Figure S4.

AGI No.	Gene name	5'-Forward-3'	5'-Reverse-3'
AT4G32540	*YUC1*	AAATGTCACCGGCAAAAGTC	GCACGTTGCTTTTGTAACCA
At4g13260	*YUC2*	GGGATGGAAGTTTGTTTAGACCTTTGC	CTGGAAACCACTTGAGCAGGC
At5g11320	*YUC4*	CCCTTCTCTCCTTTCCATCC	ACGGGGCAAAGTTTCTGAAT
At5g25620	*YUC6*	GGATCTCTGCAACTTCGGTGC	GAACATGGACAGCCCAAAAGTTGAAG
At4g28720	*YUC8*	ATCAACCCTAAGTTCAACGAGTG	CTCCCGTAGCCACCACAAG
AT1G04180	*YUC9*	CCTGCAATCAAACAGTTCTCGCGT	TGAAGCCAAGAAGGGACGTTGCTA
AT2G43010	*PIF4*	CCAGATCATCTCCGACCGGTTTG	CTAGTGGTCCAAACGAGAACCGT
At2g38120	*AUX1*	TTGGTTCAGCTGCGCATCTA	GCAGTCCAGCTTCCTAGTAA
AT1G73590	*PIN1*	ATAGTAACGACAACCAGTACG	TTGTTGGTGGCATCACCTTA
At1g70940	*PIN3*	CAAGTGGAGATTTCGGAGGA	GGCGTCTTTTGGTCTCTCTG
At1g23080	*PIN7*	TGGGCTCTTGTTGCTTTCA	AACCCCAAACTGAACATTGC
